# The human RNA surveillance factor UPF1 regulates tumorigenesis by targeting Smad7 in hepatocellular carcinoma

**DOI:** 10.1186/s13046-016-0286-2

**Published:** 2016-01-13

**Authors:** Lei Chang, Cuicui Li, Tao Guo, Haitao Wang, Weijie Ma, Yufeng Yuan, Quanyan Liu, Qifa Ye, Zhisu Liu

**Affiliations:** Department of General Surgery, Research Center of Digestive Diseases, Zhongnan Hospital of Wuhan University, Donghu Road 169, Wuhan, 430071 China; Zhongnan Hospital of Wuhan University, Institute of Hepatobiliary Diseases of Wuhan University, Transplant Center of Wuhan University, Hubei Key Laboratory of Medical Technology on Transplantation Donghu Road 169, Wuhan, 430071 China

**Keywords:** Hepatocellular carcinoma, UPF1, Smad7, Hypermethylation

## Abstract

**Background:**

In spite of progress in diagnostics and treatment of Hepatocellular Carcinoma (HCC), its prognosis remains poor, and improved treatment strategies for HCC require detailed understanding of the underlying mechanism. In this investigation we studied the role of Up-frameshift 1 (UPF1) in the tumorigenesis of HCC.

**Methods:**

We determined the expression level of UPF1 in HCC tissues with quantitative real-time PCR and western blotting and then studied its clinical significance. Sodium bisulfite sequencing was used to investigate the regulation of UPF1. We explored the biological significance of UPF1 with gain-and-loss-of-function analyses both in vitro and in vivo. The relationship between UPF1 and SMAD7 was also investigated by western blotting and immunofluorescence.

**Results:**

A great downregulation of UPF1 due to promoter hypermethylation was observed in tumor tissues compared to their adjacent normal tissues. Meanwhile, patients with low UPF1 expression have significantly poorer prognosis than those with high expression. Functionally, UPF1 regulated HCC tumorigenesis both in vitro and in vivo. Moreover, the decreased UPF1 level in HCC reduces NMD efficiency and leads to up-regulation of Smad7, then affects the TGF-β pathway.

**Conclusion:**

Our findings revealed that UPF1 is a potential tumor suppressive gene and may be a potential therapeutic target for HCC.

## Background

Hepatocellular carcinoma (HCC) is the third leading cause of cancer-related deaths worldwide [1]. Although recent advances in cancer treatment with respect to surgery, chemotherapy and biologics, majority of HCC remains incurable once it has become metastatic and has a poor prognosis [2]. The details of the molecular mechanisms underlying HCC carcinogenesis remain to be elucidated. Hepatocarcinogenesis has often been described as a multistep process involving a number of genetic alterations eventually leading to the malignant transformation of the hepatocytes [3].

Tumor suppressor genes protect normal cells from progressing to cancer in general [4]. However, these genes in cancer cells often suffer genetic mutations and aberrant epigenetic modifications [5, 6]. The mutations generate mRNA harboring premature termination codons (PTCs) that are targets of nonsense-mediated mRNA decay (NMD) pathway [7]. Hence NMD regulation and aberrant epigenetic modifications contribute to oncogenesis [8, 9] NMD is an mRNA surveillance pathway that eliminates aberrant mRNA transcript containing PTCs, and prevents the synthesis of potentially toxic truncated proteins [10]. Recent studies have shown that NMD is not only a quality control pathway, but also a regulatory pathway that controls normal gene expression. Gene expression profiling studies have shown that either loss or depletion of NMD factors in species scaling the phylogenetic scale leads to the dysregulation of 3 %–15 % of normal transcripts [11]. The core of the human NMD machinery is composed of seven polypeptides called Up-frameshift 1 (UPF1), UPF2, UPF3, SMG1, SMG5, SMG6, and SMG7 [12]. Human UPF1 also mediates two RNA decay processes, which are independent of canonical NMD as they do not require UPF2: first, it targets those mRNA molecules that are bound to the RNA binding protein Staufen1 for degradation [13]; second, together with Stem-Loop Binding Protein (SLBP), it promotes degradation of replication-dependent histone mRNAs at the end of S phase and when DNA replication is inhibited [14].

Recent studies have demonstrated that UPF1 is not only a key player in RNA degradation pathways, but that it is also essential for accomplishing DNA replication during S phase of the cell cycle [15]. UPF1 was required for S Phase progression and genome stability. UPF1 also plays a key role in the process of cell proliferation and differentiation by promoting the proliferative, undifferentiated cell state. UPF1 acts, in part, by destabilizing the NMD substrate encoding the TGFβ inhibitor, Smad7, and stimulating TGF signaling [16]. UPF1 also promotes the decay of mRNAs encoding many other proteins that oppose the proliferative, undifferentiated cell state. Liu and his colleagues found that UPF1 was down-regulated in pancreatic adenosquamous carcinoma (ASC) and the UPF1 gene is commonly mutated in pancreatic adenosquamous carcinoma [17]. The discovery of mutations in the UPF1 gene in ASC tumors represents the first known example of genetic alterations in a NMD gene in human tumors. While no studies have assessed UPF1 exact activity in human HCC, which prompted our interest in investigating its biological roles of UPF1 in hepatocarcinogenesis.

In the present study, we demonstrated that UPF1 was significantly down-regulated in HCC tissues as compared with adjacent non-tumor tissues, and this down-regulation of UPF1 was associated with decreased survival of HCC patients. Furthermore, we found that UPF1 was regulated by CpG island methylation of the putative promoter region. Functional analyses indicated UPF1 inhibited both cell growth and tumorigenicity of HCC cells, possible by targeting Smad7 and then effects on TGF-β pathways.

## Methods

### Clinical specimens and cell lines

HCC specimens and the corresponding adjacent tissues were collected from Zhongnan Hospital of Wuhan University after obtaining informed consent. The diagnosis of HCC was histopathologically confirmed. The protocols used in the study were approved by the Hospital’s Protection of Human Subjects Committee. Overall survival (OS) was defined as the interval between resection and death or the last follow-up visit. Recurrence free survival (RFS) was defined as the interval between treatment and the first diagnosis of metastasis or recurrence. L02, Huh7, Hep3B, HepG2, SMMC-7721 and HCCLM9 included in this study were purchased from the Cell Bank of Type Culture Collection (CBTCC, Chinese Academy of Sciences, Shanghai, China) and cultured in minimum essential medium (Gibco, Carlsbad, CA, USA) with 10 % fetal bovine serum (Gibco).

### Quantitative real-time PCR

Total RNA was extracted from tissues and cells using TRIzol reagent (Invitrogen, Carlsbad, CA, USA). The reverse transcription of mRNA was performed using Oligo-dT primer. Quantitative real-time PCR was performed using a standard protocol from the SYBR Green PCR kit (Toyobo, Osaka, Japan) on an iQ5 quantitative PCR System (Bio-Rad, USA). β-actin was used as an internal control, and 2^−ΔΔCT^ values were normalized to β-actin levels. Each sample was analyzed in triplicate. Primers used in this study are presented in Table 1.

**Table 1:** Primer sequence and target sequence used in this study

Gene	Sequence or Target Sequence
UPF1-F	5′-CTGCAACGGACGTGGAAATAC-3′
UPF1-R	5′-ACAGCCGCAGTTGTAGCAC-3′
Smad7-F	5′-GGACGCTGTTGGTACACAAG-3′
Smad7-R	5′-GCTGCATAAACTCGTGGTCATTG-3′
β-actin-F	5′-AGCGAGCATCCCCCAAAGTT-3′
β-actin-R	5′-GGGCACGAAGGCTCATCATT-3′
UPF1-siRNA #1	5′-GCGAGAAGGACUUCAUCAUTT-3′
	5′-AUGAUGAAGUCCUUCUCGCTT-3′
UPF1-siRNA #2	5′-GCAGCCACAUUGUAAAUCATT-3′
	5′-UGAUUUACAAUGUGGCUGCTT-3′
UPF1-siRNA #3	5′-CCUACCAGUACCAGAACAUTT-3′
	5′-AUGUUCUGGUACUGGUAGGTT-3′
UPF1-Scramble	5′-UUCUCCGAACGUGUCACGUTT-3′
	5′-ACGUGACACGUUCGGAGAATT-3′
BSP-UPF1-F	5′- GTGTTGGGATTATAGGTGTGATTAT-3′
BSP-UPF1-R	5′-CCCAACTAAAAAAACTAAACAAAAAC-3′
PcDNA3.1-UPF1-F	5′-TAATACGACTCACTATAGGG-3′
PcDNA3.1-UPF1-R	5′-CTGGAATAGCTCAGAGGC-3′

### Plasmid constructions and transfection assay

UPF1 (Gene-bank: NM_001297549.1) was cloned into pcDNA3.1 vector (Life Technology). UPF1-siRNAs were designed and synthesized by Viewsolid Biotech (Beijing, China). The sequence of siRNAs are presented in Table 1. The expression of UPF1 was confirmed by RT-qPCR and western blotting. Transfection was carried out using FuGene HD transfection reagent (Roche, Indianapolis, IN) according to the manufacturer’s protocol.

### Immunohistochemistry (IHC) and Immunofluorescence (IF)

For IHC, paraffin sections were cut to a thickness of 4 μm, the slides were deparaffinized in xylene and rehydrated with ethanol, and the endogenous peroxidase was inactivated with 0.3 % hydrogen peroxide. All of the steps were performed using an UltraSensitiveTM S-P kit (Maixinbio, China) according to the manufacturer’s protocol. The total immunostaining score was calculated as the sum of the positive percentage and the staining intensity of the stained cells, which ranged from 0 to 6. The percent positivity was scored as “0” (0–25 %), “1” (26–50 %), “2” (51–75 %), and “3” (≥75 %). The staining intensity was scored as “0” (no staining), “1” (weakly stained), “2” (moderately stained), and “3” (strongly stained). A negative expression of protein was defined as a total score ≤ 3, and a positive expression was defined as a total score ≥ 4. For immunofluorescence, cells were fixed in 4 % paraformaldehyde, permeabilized using 0.5 % Triton X-100 and incubated with primary antibody and secondary antibodies used according to the manufacturer’s protocol. The coverslips were counterstained with DAPI and imaged with a confocal laser-scanning microscope (Olympus FV1000, Tokyo, Japan). Antibodies used in this study were presented in Table 2.

**Table 2:** Antibody information used in this study

Antibody	WB	IHC	IF	Specificity	Company
GAPDH(KM9002)	1:5000			Mouse monoclonal	Sungene
UPF1(#12040)	1:1000	1:100	1:400	Rabbit polyclona	Cell Signaling Technology
SMAD7(sc-101152)	1:500	1:200	1:100	Mouse monoclonal	Santa Cruz Biotechnology

### Flow cytometry analysis

Cells transiently transfected with siRNA or vector were harvested 48 h after transfection. Following the double staining with Annexin V FITC and Propidium Iodide (PI), the cells were analyzed by flow cytometry (FACScan®; BD Biosciences, San Jose, CA) equipped with CellQuest software (BD Biosciences). For the cell cycle analysis, the cells were stained with PI using the CycleTESTTM PLUS DNA reagent kit (BD Biosciences) according to the manufacturer’s instruction.

### Western blotting

Tissular and cellular proteins from each sample were electrophoresed by SDS-polyacrylamide gel electrophoresis (4 % stacking and 10 % separating gels), then transferred onto polyvinylidene fluoride PVDF membranes (Millipore, USA), then incubated with primary antibodies overnight at 4 °C. After the incubation with secondary antibodies, the PVDF membranes were subsequently subjected to immunoblotting analysis using the ECL immunoblotting kit (Beyotime Institute of Biotechnology, China) according to the manufacturer’s protocol. Antibodies information used in this study was listed in Table 2.

### Transwell and wound healing assay

The invasion of cells was assessed using Matrigel-coated chambers with 8 μm pores (BD Biosciences, Franklin Lakes, NY, USA). Briefly, hepatoma cells (1 × 10^5^) were seeded in serum-free medium and were allowed to translocate toward complete media supplemented with 10 % fetal bovine serum after depleted of UPF1. The cells that had invaded through the membrane to the lower surface were fixed, stained and counted after 24 h. HCC cells (1 × 10^6^ cells/well) were treated with the indicated reagents, and wounds were made using a 100 μl plastic pipette tip. The size of the wound was measured after 24 h of wound formation and photographed.

### CCK-8 cell proliferation assay

Cell proliferation assay was performed by using Cell Counting Kit-8 (Dojindo, Japan). Briefly, cells were plated in 96-well plates in triplicate at aproximately 3–5 × 10^4^ cells per well and cultured in the growth medium. Cells were then treated with the indicated reagent and the numbers of cells per well were measured by the absorbance (450 nm) at the indicated time points.

### Sodium bisulfite sequencing

MethPrimer (http://www.urogene.org/cgi-bin/methprimer/methprimer.cgi) was used to analyze the CpG islands. Genomic DNA was extracted using DNeasy Tissue Kit (QIAGEN, Valencia, CA). Genomic DNA was treated with sodium bisulfite using the CpGenome DNA Modification Kit (Serologicals Corp, Norcross, GA) according to the manufacturer’s protocol. PCR products were subcloned, and ten constructs representing each region from each sample were randomly selected for sequence analysis. DNA methylation data were analyzed by using BiQ Analyzer (http://biq-analyzer.bioinf.mpi-inf.mpg.de/.).

### Tumor formation assay in a nude mouse model

HCCLM9 cells stably transfected with pcDNA3.1-UPF1 or empty vector were suspended at 1 × 10^7^ cells/ml. A total of 100 μl of suspended cells were subcutaneously injected into the male athymic BALB/c nude mice (5 weeks old). Tumor volumes and weights were measured beginning from day 7 after the tumor cell injection.

### Tail vein injections into athymic mice

HCCLM9 cells stably transfected with pcDNA3.1-UPF1 or the empty vector were suspended at 2 × 10^7^ cells/ml. Suspended cells (100 μl) were injected into the tail veins of 10 mice (5 weeks old), which were sacrificed 7 weeks after injection. The lungs were removed and visible tumors on the lung surface were counted and used for further analysis.

### Statistical analysis

Data are presented as the means ± standard deviation from at least three independent experiments. Student’s t-test, χ^2^ test or Fisher’s exact tests were used for comparisons between groups with SPSS 22.0 software (IBM, Chicago, IL, USA). The Kaplan-Meier test was used to estimate OS and RFS. A value of *p* < 0.05 was considered significant.

## Results

### UPF1 was strongly downregulated in HCC and associated with HCC progression

**Fig. 1:**
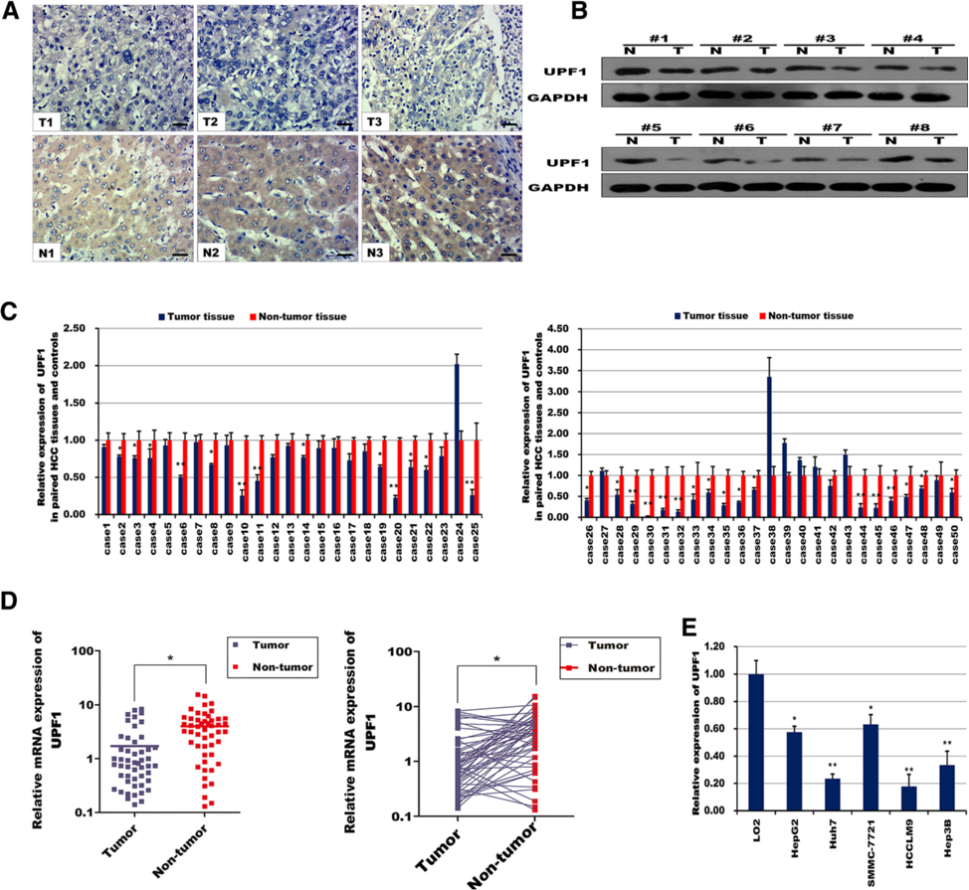
UPF1 was downregulated in human HCC tissues and HCC cells. **a** Representative IHC images showing the downregulation of UPF1 in HCC tissue (up) and the normal expression of UPF1 in HCC adjacent non-tumor tissue (down). (Scale bars = 50 μm) (**b**) The UPF1 protein expression levels in HCC tissues and paired non-tumor tissues were examined using Western blotting. **c** The expression of mRNA level of UPF1 was detected in 50 HCC patients by RT-qPCR. Data was presented as fold change of HCC tissues relative to adjacent normal regions. *p < 0.05; **p < 0.01. **d** Relative UPF1 expression level in HCC tissues and adjacent normal regions. *p < 0.01. **e** The relative mRNA level of UPF1 in HCC cell lines (HepG2, Huh7, SMMC-7721, HCCLM9, and Hep3B) and human normal liver cell (L02); for all quantitative results, the data are presented as the mean ± SEM, and the error bars represent the standard deviation obtained from three independent experiments. *p < 0.05; **p < 0.01

**Fig. 2:**
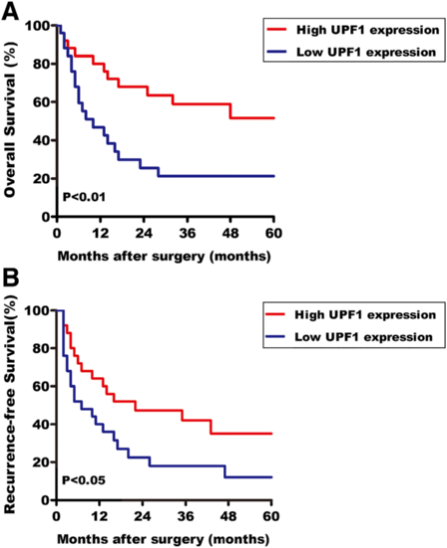
Low UPF1 expression predicts poor prognosis in patients with HCC. **a** Kaplan-Meier curves displaying overall survival of patients with high UPF1 expression vs low UPF1 expression (*p* < 0.01, Log-rank Test). **b** Kaplan-Meier curves displaying recurrence-free survival of patients with high UPF1 expression vs low UPF1 expression (*p* < 0.05, Log-rank Test). The median mRNA expression level was used as the cutoff. Patients with UPF1 mRNA expression values below the 50th percentile were classified as having lower UPF1 levels. Patients with UPF1 expression values above the 50th percentile were classified as having higher UPF1 levels

The expression level of UPF1 was first examined by IHC in 50 HCC patients. UPF1 was downregulated in HCC tissues compared with adjacent non-tumor tissues (Fig. 1a). Western blotting further confirmed the lower expression of UPF1 in HCC tissues (Fig. 1b). We next examined the expression of UPF1 by quantitative RT-PCR in 50 HCC patients. Figure 1c and d show significantly lower UPF1 expression in HCC tissues. In vitro, UPF1 expression is decreased in hepatoma cells compared with normal liver cells (Fig. 1e). Aberrant expression of UPF1 was closely correlated with Edmondson-Steiner grade (*P* = 0.015), Barcelona Clinic Liver Cancer stage (BCLC; *P* = 0.020) and portal vein tumor thrombus (PVTT; *P* = 0.031) (Table 3). Furthermore, Kaplan-Meier and log-rank test analyses suggested that HCC patients with low UPF1 expression have shorter overall survival (OS) and higher recurrence rates than those with high expression of UPF1 (Fig. 2a and b).

**Table 3:** Relationship between UPF1 expression and clinicopathologic parameters of HCC patients

Characteristics	Number of cases	UPF1 expression		P value
		Low(n = 25)	High(n = 25)	
Age(years)				0.152
≥ 65	29	17	12	
< 65	21	8	13	
Gender				0.157
Male	40	18	22	
Female	10	7	3	
Tumor size				0.364
≥ 5 cm	32	19	13	
< 5 cm	18	6	12	
Edmondson-Steiner				0.015*
I-II	34	21	13	
III-IV	16	4	12	
HBV infection				0.088
Postive	39	17	22	
Negtive	11	8	3	
BCLC stage				0.020*
A	42	24	18	
B + C	8	1	7	
Liver cirrhosis				0.225
Yes	34	15	19	
No	16	10	6	
Serum AFP(μg/L)				0.771
≥ 400	31	16	15	
< 400	19	9	10	
Tumor number				0.747
Singular	37	18	19	
Multifocal	13	7	6	
PVTT				0.031*
Yes	15	4	11	
No	35	21	14	

Barcelona Clinic Liver Cancer stage: BCLC

Portal vein tumor thrombus: PVTT

*p < 0.05

### UPF1 suppressed HCC tumorigenesis in vitro and in vivo

**Fig. 3:**
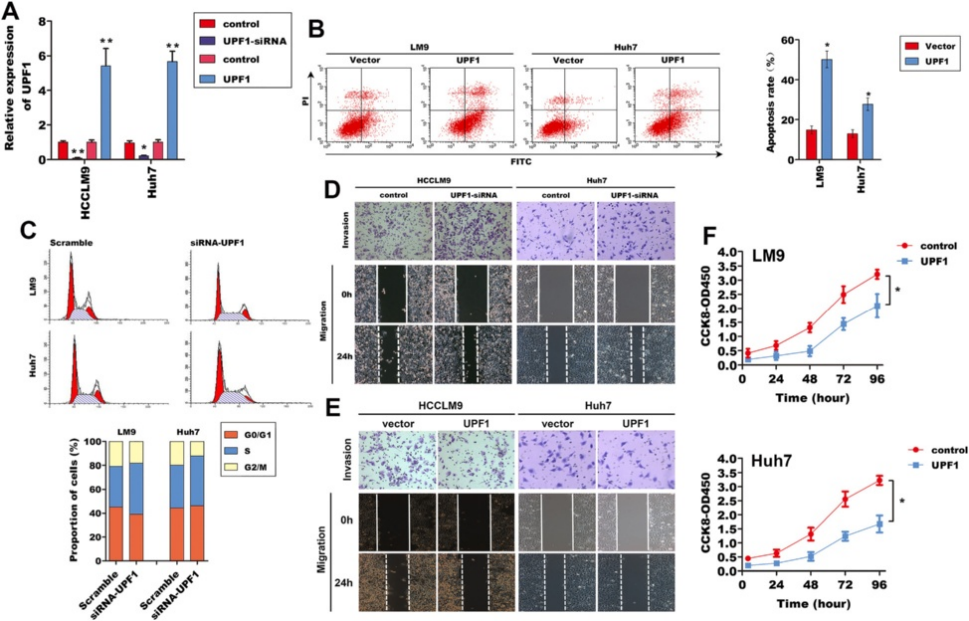
The functional analysis of UPF1 in HCC cells. **a** UPF1 mRNA level was detected in HCCLM9 and Huh7 cells after treatment with UPF1-siRNA and pCDNA3.1-UPF1 by RT-qPCR. *p < 0.05; **p < 0.01 (**b**) Flow cytometry was applied to examine the apoptosis of HCCLM9 and Huh7 cells after treatment with pCDNA3.1-UPF1 and stained with apoptosis markers (FITC-Annexin V and PI). In the apoptosis map, FITC-Annexin V^+^/PI^+^ indicates late apoptosis, FITC-Annexin V^+^/PI^−^ indicates early apoptosis, and FITC-Annexin V^−^/PI^−^ indicates normal live cells. *p < 0.05 (**c**) Flow cytometry analysis showing significant decreases of cells in the S- phase, when UPF1 was silenced in HCCLM9 and Huh7 cells. **d** The ability of cancer cell invasion and migration was measured by using transwell and wound-healing assays when UPF1 was silenced in HCCLM9 and Huh7 cells (Transwell [original magnification, ×200]; wound-healing assays [original magnification, ×100]). **e** The ability of cancer cell invasion and migration was measured by using transwell and wound-healing assays when UPF1 was overexpressed in HCCLM9 and Huh7 cells (Transwell [original magnification, ×200]; wound-healing assays [original magnification, ×100]). **f** CCK-8 and assay showing that overexpression of UPF1 inhibited the proliferation of HCCLM9 and Huh7 cells. *p < 0.05

The significant downregulation of UPF1 expression in HCC tissues suggested possible biological significance in tumorigenesis. We evaluated the effects of UPF1 on cell apoptosis and cell cycle progression in HCCLM9 and Huh7 cells transfected with either pcDNA3.1-UPF1 or UPF1-siRNA. Measured using RT-qPCR, the expression of UPF1 was 6.3-fold (HCCLM9) and 5.7-fold (Huh7) increased in cells transfected with pcDNA3.1-UPF1, but 20-fold (HCCLM9) and 5-fold (Huh7) deceased in cells transfected with 20nM UPF1-siRNA (Fig. 3a). Cells treated with empty vector or scrambled siRNA showed no significant differences in UPF1 expression.

Fluorescence-activated cell sorting (FACS) analysis showed significant increases in apoptotic cells when UPF1 was overexpressed (Fig. 3b). Cell cycle analysis showed that in UPF1-siRNA transfected cells, the percentage of cells in the S phase was more than that of scramble siRNA transfected cells. (Fig. 3c). Similar results were observed in Huh7 cells. Together, these results suggest that UPF1 could suppress HCC cell proliferation by inducing cell cycle arrest.

To further examine the effects of UPF1 on HCC tumorigenesis, the biological consequences of UPF1 in regulating cancer cell invasion and migration were examined using cell biology assays. As shown in Fig. 3d, functionally, knockdown UPF1 increased the cell invasion and migration of HCC cells. While, overexpression of cellular UPF1 significantly decreased the cell invasion, migration and proliferation of HCC cells (Fig. 3e and f).

**Fig. 4:**
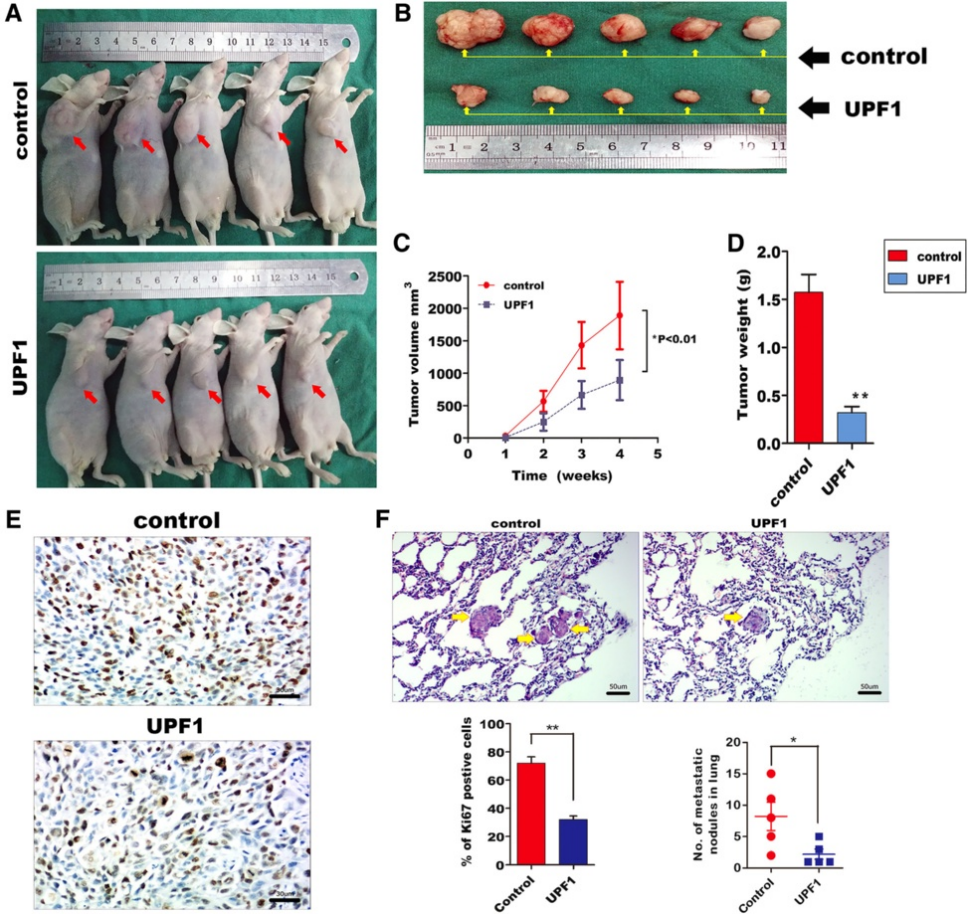
Downregulation of UPF1 promoted tumor growth and metastasis in vivo. **a** Representative images of tumors formed in nude mice injected subcutaneously with HCCLM9 control cells (Control) and pcDNA3.1-UPF1 stable clones (UPF1). **b** Representative images of tumors removal from the nude mice. **c**, **d** Effect of UPF1 on HCC growth was described by the tumor growth curve and tumor weight in the two groups. Data are shown as the mean ± s.d. *p < 0.05; **p < 0.01. **e** Immunohistochemical staining for Ki-67 in tumor xenografts of nude mice formed by the two groups. (Scale bars = 30 μm). The proportion of Ki-67 positive cells was also shown. **p < 0.01. **f** Representative images of HE staining of metastatic nodules in the lungs of nude mice. The metastatic nodules are indicated by yellow arrows. (Scale bars = 50 μm). *p < 0.05

To investigate the roles of UPF1 in tumorigenesis in vivo, HCCLM9 cells which were stably transfected with pcDNA3.1-UPF1 and control cells were inoculated into the oxter of male nude mice. We found UPF1 overexpressed dramatically inhibited tumor growth (Fig. 4a-d). Moreover, UPF1 overexpressed dramatically reduced positivity for Ki-67 (Fig. 4e). We next evaluated the in vivo effects of UPF1 on metastasis using a lung metastasis model. The numbers of pulmonary metastatic nodules in the lung were clearly greater in the control group compared with the UPF1 overexpressed group. In addition, the HE staining of lung section showed that UPF1 overexpressed reduced the number of visible lung metastases (Fig. 4f).

### CpG hypermethylation downregulated UPF1 expression in HCC

**Fig. 5:**
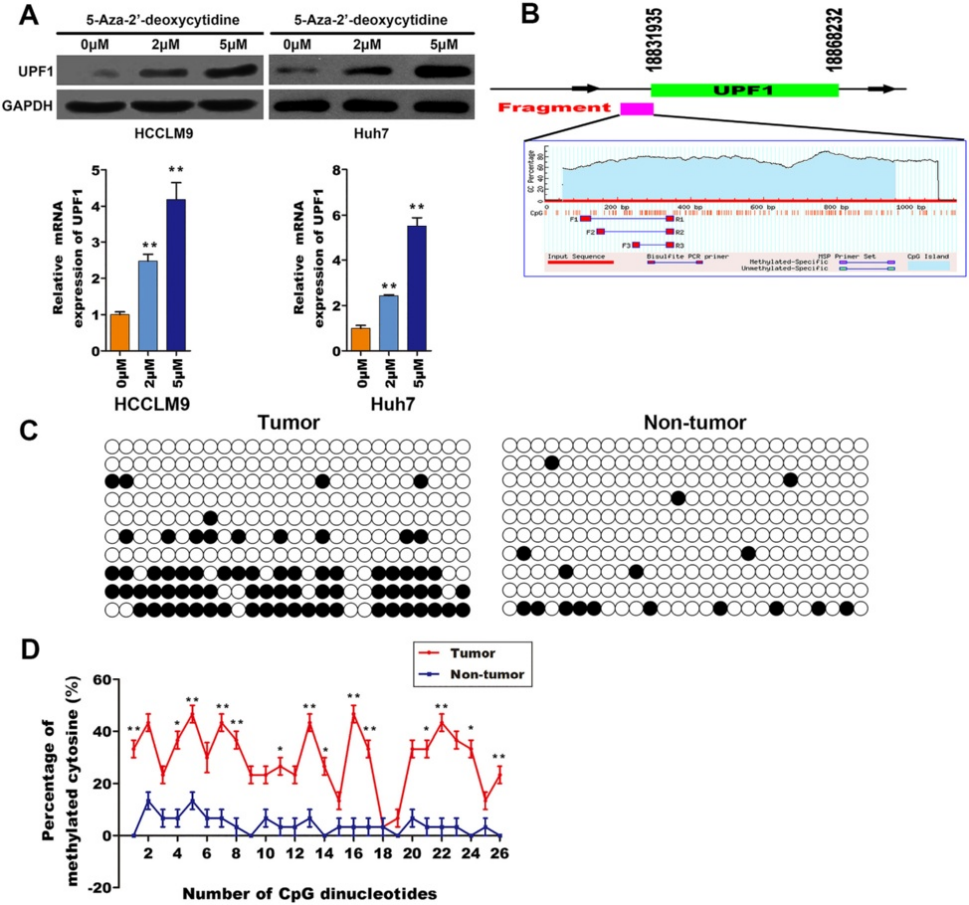
CpG island hypermethylation was associated with UPF1 downregulation. **a** Demethylation following treatment with 5-Aza-2′ -deoxycytidine induced UPF1 up-regulation in HCCLM9 and Huh7 cells, the protein and mRNA expression level of UPF1 was checked by western blotting and RT-qPCR respectively. **p < 0.01. (**b**) The CpG island enriched sites in the upstream region of UPF1 were analyzed using CpG islandsearcher. **c** The CpG island enriched sites in the upstream region of UPF1 were analyzed using Meth-Primer (**d**) Bisulfite sequencing of the putative promoter region. Each line represents one PCR product, and ten PCR products are shown for each sample. ●, methylated CpGs; ○, unmethylated CpGs. **e** The methylation status of each CpG in this region was quantified using the percentage of methylated CpGs among all PCR products. *p < 0.05, **p < 0.01

Study had shown hypermethylation of tumor suppressor genes contribute to tumorigenesis [18]. To investigate the underlying mechanism responsible for downregulation of UPF1 in HCC, we tested whether hypermethylation are involved in the progression. Using MethPrimer software we found several CpG islands were contained in promoter region of UPF1. We speculated hypermethylation may take a key role in the regulation of UPF1. First, 5-Aza-2′-deoxycytidine-the methylation inhibitor was used to demethylate genomic DNA. As shown in Fig. 5a, the protein and mRNA expression level was gradually increased in HCCLM9 and Huh7 cells following treatment with increased concentrations of 5-Aza-2′-deoxycytidine. To explore the relationship between promoter methylation and UPF1 gene downregulation, we utilized CpG island prediction software to analyze the promoter region of UPF1. As shown in Fig. 5b fragment (18831624 ~ 18832749 in chromosome 19, NC_000019.10) was rich in CpG dinucleotides and predicted as a putative promoter region by the Meth-Primer software. To examine the methylation pattern, the fragment containing a total of 26 CpG dinucleotides, was amplified from genomic DNA isolated from HCC and non-tumor tissues treated with either sodium bisulfite or vehicle. Figure 5c shows a representative methylation pattern of the 26 CpGs putative promoter region. In non-tumor tissues, the majority of CpGs were unmethylated, whereas in the tumor tissues, most of the CpGs were methylated. The methylation status of each CpG was quantified using the percentage of methylated CpGs among all PCR products analyzed. 53.85 % (14/26) CpGs were hypermethylated in tumors compared to 15.38 % (4/26) in normal tissues (Fig. 5d).

### Smad7 was strongly up-regulated in HCC

**Fig. 6:**
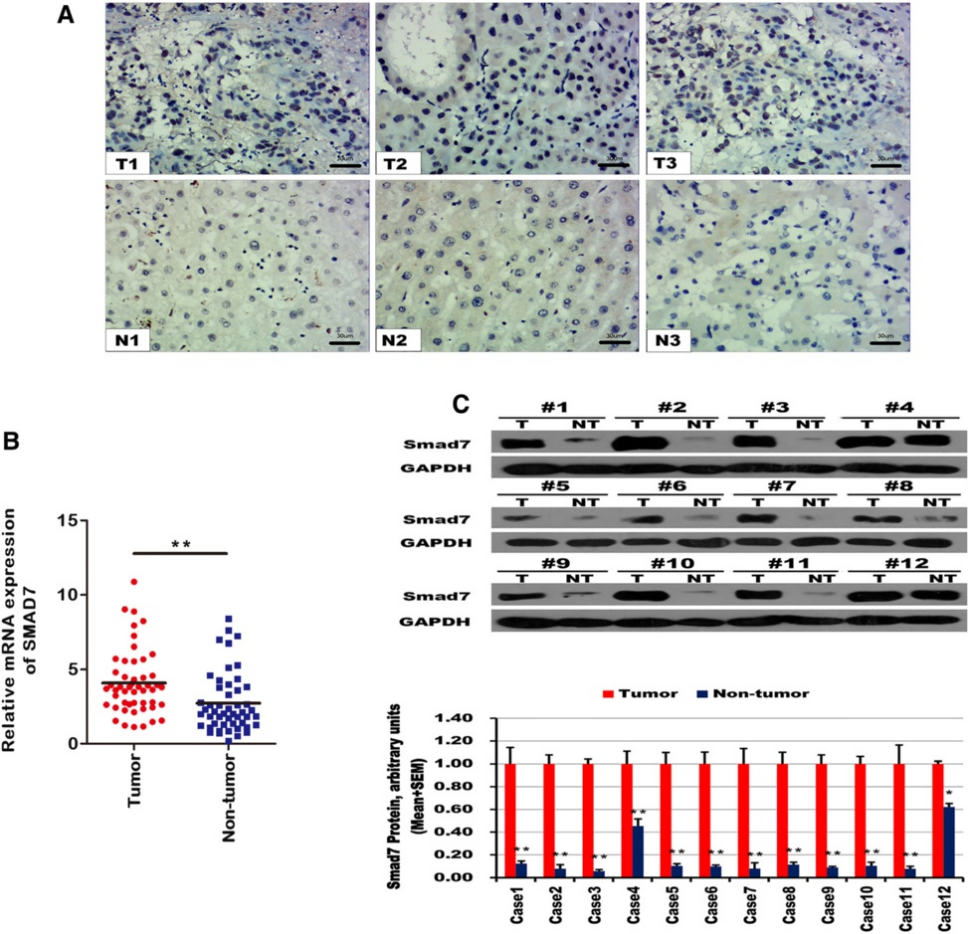
Smad7 was overexpressed in HCC tissue. **a** Representative IHC images showing the upregulation of Smad7 in HCC tissue (up) and the normal expression of Smad7 in HCC adjacent non-tumor tissue (down). (Scale bars = 30 μm). **b** The expression of mRNA level of Smad7 was detected in 50 HCC patients by RT-qPCR. **p < 0.01. **c** The Smad7 protein expression levels in HCC tissues and paired non-tumor tissues were examined using western blotting. *p < 0.05; **p < 0.01

The expression level of Smad7 was first examined by IHC in 50 HCC patients. Smad7 was up-regulated in HCC tissues compared with adjacent non-tumor tissues (Fig. 6a). We next examined the expression of UPF1 by quantitative RT-qPCR in 50 HCC patients. Figure 6b showed significantly higher Smad7 expression in HCC tissues. Western blotting further confirmed the higher expression of Smad7 in HCC tissues (Fig. 6c).

### UPF1 suppressed HCC tumorigenesis by targeting Smad7 and affected TGF-β pathway

**Fig. 7:**
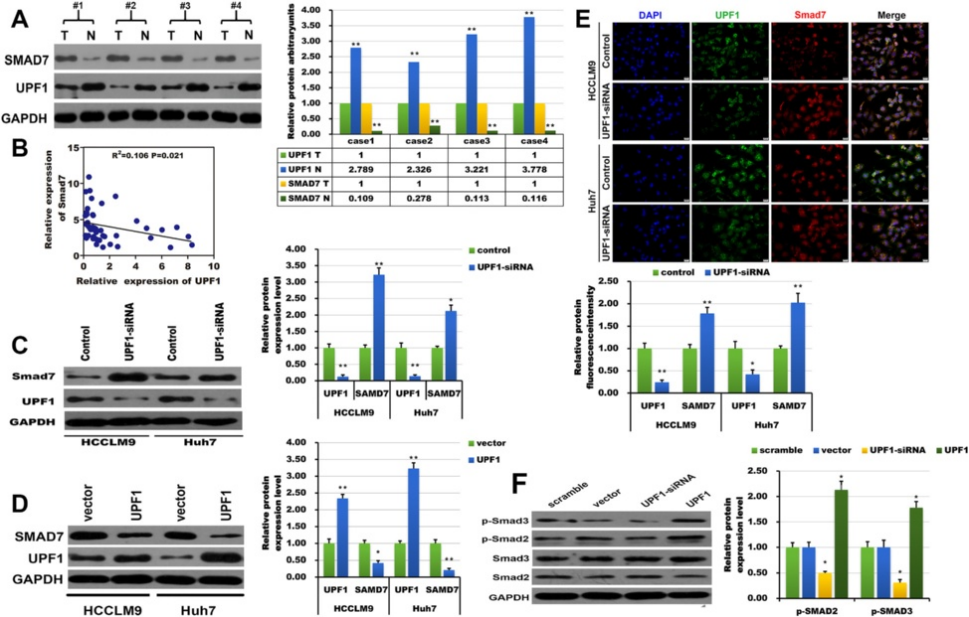
UPF1 affected the TGF-β pathway by targeting Smad7. **a** The expression of UPF1 and Smad7 protein was illustrated in paired tumor tissues and non-tumor tissues from patients with HCC. N indicates adjacent normal tissue; T, tumor tissue. (**b**) Bivariate correlation analysis of the negative association between UPF1 mRNA expression level and Smad7 mRNA expression level in 50 HCC tissues. *p < 0.05. (**c**, **e**) The UPF1 and Smad7 protein expression levels in HCC cells were examined using western blotting and immunofluorescence when UPF1 was silenced. *p < 0.05; **p < 0.01, (Scale bars =20 μm). **d** The UPF1 and Smad7 protein expression levels in HCC cells were examined using western blotting when UPF1 was overexpressed. *p < 0.05; **p < 0.01. **f** The expression and phosphorylation of key molecules of the TGF-β pathways (Smad2, Smad3) were detected using western blotting. GAPDH was used as an internal control. *p < 0.05

Study had shown that UPF1 could promote TGF-β signaling and inhibits neural differentiation by targeting Smad7 mRNA [16]. Whether UPF1 affects Smad7 in human tumorigenesis? Up to now, it has not been reported, which prompted our interest in investigating the relationship between UPF1 and Smad7 in HCC. First, we analyzed the relationship between their expression levels in HCC tissues. As shown in Fig. 7a and b, the protein expression level of UPF1 and Smad7 in the paired tissues was negative associated and an inverse correlation was found between UPF1 and Smad7 expression levels in 50 pairs of HCC tissues by RT-qPCR. Western blotting and immunofluorescence confirmed that Smad7 expression level was up-regulated when UPF1 was silenced in HCC cells (Fig. 7c and e). While the Smad7 expression level was down-regulated when UPF1 was overexpressed in HCC cells (Fig. 7d). Previous studies had demonstrated the key role of Smad7 in the TGF-β pathway. As shown in our study, UPF1 could affect the expression of Smad7 in HCC. Therefore, we speculated that UPF1 could affect the TGF-β pathway. To verify these findings, the key molecules in the TGF-β pathway (Smad2 and Smad3) were detected by western blotting. As shown in Fig. 7f, UPF1 overexpression significantly increased phosphorylation of Smad2/3, whereas total Smad2/3 expression level was not significantly altered. These results were reversed under conditions of UPF1 knockdown. These results indicate that the TGF-β pathways may be involved in UPF1 suppression of tumorigenesis.

## Discussion

In this study, we reported the correlation between UPF1 silencing and HCC. Immunohistochemical analysis, western blotting and RT-qPCR experiments showed that the down-regulation of UPF1 was associated with malignant progression of HCC. Epigenetic silencing of UPF1 gene was observed in low UPF1-expressing HCC cell lines. Therefore, we deducted that UPF1 silencing may be required for tumorigenesis and has a potential to be developed as a biomarker for malignant phenotype of HCC, which possesses considerable value in prognosis prediction, progression monitoring and treatment evaluating. For future application, it is critical to design reasonable clinical trial for evaluation of this biomarker. Not only overall survival and recurrence-free survival should be evaluated in randomized, controlled trials, some key factors with regard to HCC, such as HBV infection information, will be considered.

Generally speaking, hypermethylation of tumor suppressor genes and demethylation of oncogenes contribute to tumorigenesis [18, 19]. Cancer-linked hypermethylation and hypomethylation of gene promoter is often associated with cell proliferation and differentiation [20]. In this study, we found UPF1 is a potential tumor suppressor, which negatively regulates proliferation of cancer cells, we also observed that UPF1 is down-regulated in HCC tissues. In HCC tissues and cell lines, the methylation states of the CpGs in UPF1 promoter region were found to be hypermethylated. Moreover, UPF1 gene was also re-expressed following treatment with 5-Ad, a potent demethylating agent, suggesting that status of DNA demethylation was important for the active expression of UPF1.

NMD is an evolutionally conserved mRNA quality-control mechanism that selectively degrades aberrant mRNA containing premature termination codons in order to prevent the accumulation of truncated proteins [21], which are often non-functional or potentially deleterious in the cells. Three conserved proteins UPF1, UPF2, and UPF3 make up the key NMD machinery with UPF1 as the important member in this protein set [22–24] UPF1 acts in concert with the peptide release factors eRF1/eRF3 to recognize aberrant translation termination events and, together with UPF2 and UPF3, triggers degradation of mRNA in a subsequent step [25, 26]. In addition to its role in NMD, UPF1 also regulates mRNAs in a NMD-independent manner. For example, UPF1 is recruited by the RNA-binding protein Staufen to the downstream of a stop cordon to degrade some mRNAs in a Staufen-mediated decay [13]. Human UPF1 has been shown to regulate the degradation of histone mRNAs in mammalian cells [27]. Moreover, UPF1 is involved in nonsense-mediated altered splicing [28]. Although NMD pathway has been extensively studied, the regulatory mechanism of NMD in cancer is still not well understood. The first report about the relationship between UPF1 and human tumor is pancreatic adenosquamous carcinoma. UPF1 was found down-regulated in pancreatic adenosquamous carcinoma [17], here, in our study, we found UPF1 was also down-regulated in HCC. In addition, as a key molecule in the TGF-β pathway, Smad7 was an inhibitory SMAD protein. We found UPF1 could suppress the Smad7 level in HCC and then affected the TGF-β pathway.

## Conclusion

In conclusion, the current study revealed that UPF1 was down-regulated in HCC cells and tissues. Furthermore, UPF1 suppressed tumorigenesis of HCC. Finally, the reduced UPF1 expression, due to promoter hypermethylation, attenuated NMD, leaded to the dysregulation of Smad7, which indicated aberrant mRNA surveillance mechanism in HCC. All the results indicated that UPF1 played an important role during HCC carcinogenesis and may serve as a putative target for HCC diagnosis and therapy.
